# The endosomal trafficking regulator LITAF controls the cardiac Nav1.5 channel via the ubiquitin ligase NEDD4-2

**DOI:** 10.1074/jbc.RA120.015216

**Published:** 2021-01-13

**Authors:** Nilüfer N. Turan, Karni S. Moshal, Karim Roder, Brett C. Baggett, Anatoli Y. Kabakov, Saroj Dhakal, Ryota Teramoto, David Yi-Eng Chiang, Mingwang Zhong, An Xie, Yichun Lu, Samuel C. Dudley, Calum A. MacRae, Alain Karma, Gideon Koren

**Affiliations:** 1 Cardiovascular Research Center, Division of Cardiology, Department of Medicine, Rhode Island Hospital, Warren Alpert Medical School, Brown University, Providence, Rhode Island, USA; 2 Physics Department and Center for Interdisciplinary Research on Complex Systems, Northeastern University, Boston, Massachusetts, USA; 3 Cardiovascular Division, Brigham and Women's Hospital, Harvard Medical School, Boston, Massachusetts, USA; 4 Cardiovascular Division, Department of Medicine, University of Minnesota, Minneapolis, Minnesota, USA

**Keywords:** action potential duration, cardiomyocyte, LITAF, Nav1.5, NEDD4-2, sodium channel, ubiquitin, zebrafish, E3 ubiquitin ligase, computer modelling

## Abstract

The QT interval is a recording of cardiac electrical activity. Previous genome-wide association studies identified genetic variants that modify the QT interval upstream of *LITAF* (lipopolysaccharide-induced tumor necrosis factor-α factor), a protein encoding a regulator of endosomal trafficking. However, it was not clear how LITAF might impact cardiac excitation. We investigated the effect of LITAF on the voltage-gated sodium channel Nav1.5, which is critical for cardiac depolarization. We show that overexpressed LITAF resulted in a significant increase in the density of Nav1.5-generated voltage-gated sodium current *I*_Na_ and Nav1.5 surface protein levels in rabbit cardiomyocytes and in HEK cells stably expressing Nav1.5. Proximity ligation assays showed co-localization of endogenous LITAF and Nav1.5 in cardiomyocytes, whereas co-immunoprecipitations confirmed they are in the same complex when overexpressed in HEK cells. *In vitro* data suggest that LITAF interacts with the ubiquitin ligase NEDD4-2, a regulator of Nav1.5. LITAF overexpression down-regulated NEDD4-2 in cardiomyocytes and HEK cells. In HEK cells, LITAF increased ubiquitination and proteasomal degradation of co-expressed NEDD4-2 and significantly blunted the negative effect of NEDD4-2 on *I*_Na_. We conclude that LITAF controls cardiac excitability by promoting degradation of NEDD4-2, which is essential for removal of surface Nav1.5. LITAF-knockout zebrafish showed increased variation in and a nonsignificant 15% prolongation of action potential duration. Computer simulations using a rabbit-cardiomyocyte model demonstrated that changes in Ca^2+^ and Na^+^ homeostasis are responsible for the surprisingly modest action potential duration shortening. These computational data thus corroborate findings from several genome-wide association studies that associated LITAF with QT interval variation.

The voltage-gated sodium channel Nav1.5 is responsible for the initial upstroke of cardiac action potential (1, 2). Post-translational modifications such as phosphorylation or ubiquitination are essential for correct expression and function of Nav1.5 (1). The activity and density of Nav1.5 channels at the membrane depend on forward trafficking, stability, and domain-targeting mediated by anchoring proteins and retrograde trafficking (3). Retrograde trafficking of cardiac Nav1.5 depends on the E3 ubiquitin ligase NEDD4-2 (neural precursor cell-expressed developmentally down-regulated 4 type 2), which accelerates the degradation of Nav1.5 by ubiquitination (4, 5). NEDD4-2 is highly expressed in the heart (6), where it down-regulates Nav1.5 through P*X*Y motif recognition by its WW domains (5). However, the details of this regulation are not well-understood.

Genetic modifications in the *SCN5A* gene, which encodes Nav1.5, cause inherited long QT syndrome 3, Brugada syndrome, atrial fibrillation, sick sinus syndrome, progressive cardiac conduction defect, or dilated cardiomyopathy (reviewed by Li *et al.* (7)). Also, dysfunction of Nav1.5 in myocardial ischemia and heart failure is proarrhythmic (2, 8, 9, 10). Several genome-wide association studies for loci that modify the QT interval and the risk of sudden cardiac death (11, 12, 13) have identified three SNPs located in or near genes encoding proteins involved in ubiquitination (RNF207, RFFL, and LITAF) (14, 15). LITAF (lipopolysaccharide-induced tumor necrosis factor-alpha factor) is a regulator of endosomal trafficking (16, 17, 18) and inflammatory cytokines (19, 20, 21) and an adapter molecule for members of the NEDD4 (neural precursor cell-expressed developmentally down-regulated protein 4)–like family of E3 ubiquitin ligases (17, 22). The N terminus of LITAF contains two P*X*Y motifs, which are essential for interacting with members of the NEDD4 family of HECT (homologous to the E6-AP C terminus) domain ubiquitin ligases via their WW domains (17, 22, 23). LITAF also interacts with members of the ESCRT (endosomal sorting complex required for transport) family, including TSG101 (tumor susceptibility gene 101) and STAM1 (signal-transducing adaptor molecule 1), recruiting them to the early endosomal membrane and controlling endosome-to-lysosome trafficking and exosome formation (16, 17, 18). Mutations clustered around the hydrophobic region required for membrane localization in LITAF cause Charcot–Marie–Tooth disease, an inherited peripheral neuropathy. They also result in mislocalization and impaired endosome-to-lysosome trafficking of membrane proteins (16, 24). Importantly, the genetic variant rs8049607 located within an intergenic enhancer region (25) is associated with a very modest QT interval prolongation of 1.2 ms (11, 12, 13). This variant (rs8049607) is associated with reduced LITAF mRNA transcript levels in the left ventricle (26, 27). Thus, a reduction in LITAF prolongs the QT interval.

Based on the genome-wide association studies' findings (11, 12, 13) and LITAF's functional role in endosome-to-lysosome trafficking, we hypothesized that LITAF is a candidate for regulation of cardiac excitation, likely acting as an effector of ion channel complex trafficking or degradation. Indeed, we have recently shown that LITAF acts as an adaptor protein promoting NEDD4-1–mediated ubiquitination and subsequent degradation of L-type calcium channels (LTCCs), and gain of function of LITAF is associated with shortening of action potential duration (APD) (27). In this study, we present data that support an additional role for LITAF in modulating membrane density and function of cardiac Nav1.5 via the ubiquitin ligase NEDD4-2. Uniquely, gain of function of LITAF increases the expression of sodium channels in the membrane.

## Results

### The voltage-gated sodium current I_Na_ is regulated by LITAF in 3-week-old rabbit cardiomyocytes

**Figure 1:**
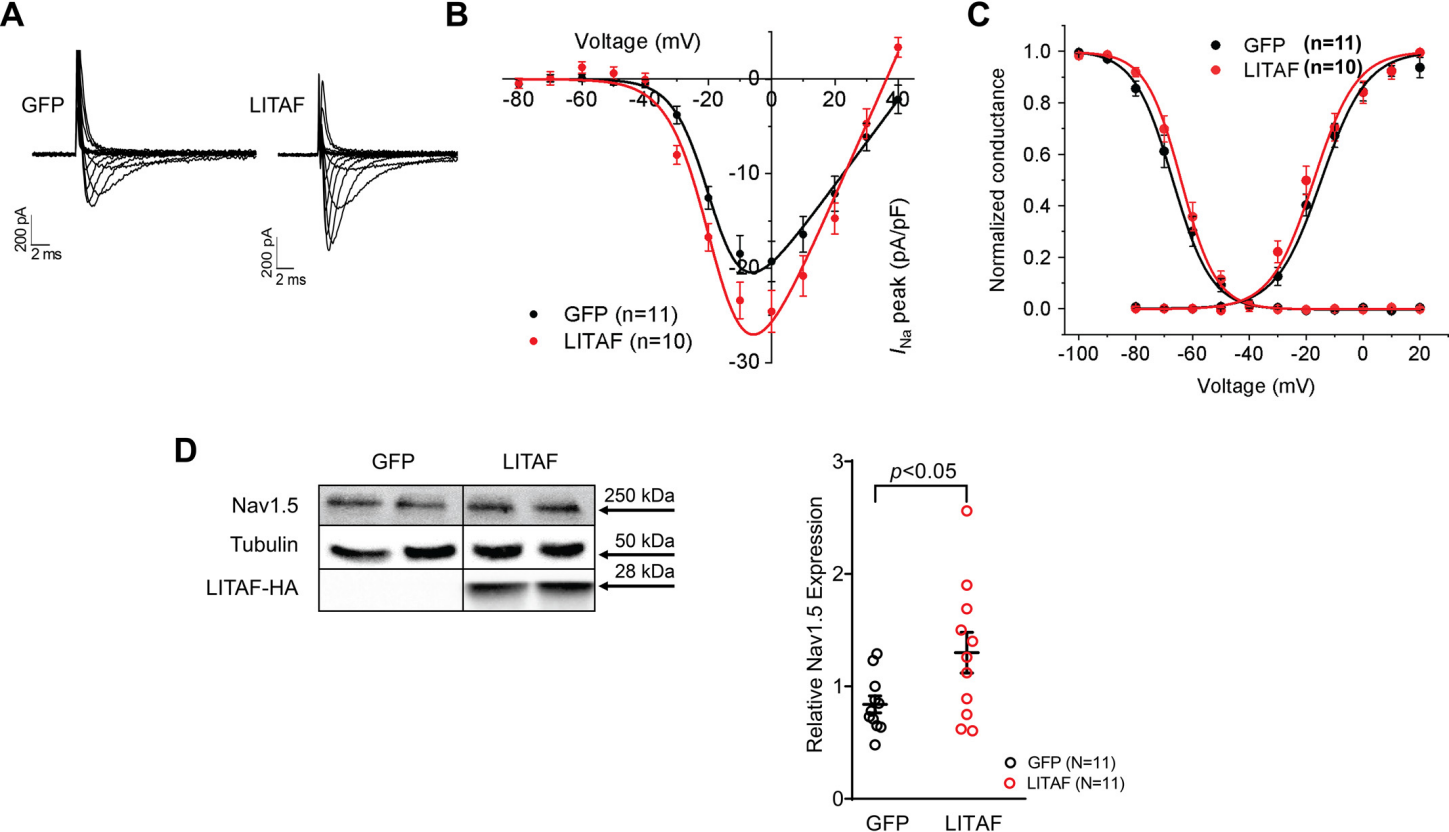
**LITAF increases *I*_Na_ and Nav1.5 levels in 3wRbCM.** Cardiomyocytes were transduced with adenovirus encoding GFP or LITAF-HA (MOI of 10, 48 h). *A*, *left panel*, representative traces of *I*_Na_ in control, GFP-expressing 3wRbCM. *I*_Na_ was activated with depolarizing membrane potentials between −80 and +40 mV from −100 mV holding potential. *Right panel*, typical *I*_Na_ traces in GFP- and LITAF-expressing 3wRbCM. *B*, voltage dependence of *I*_Na_ current in transduced cardiomyocytes. The currents were normalized to cell capacitance (means ± S.E.; *p* < 0.01, 3wRbCM from three rabbits were used for both LITAF and GFP experiments). *C*, activation curves of *I*_Na_ were obtained from data shown in *B*, whereas inactivation curves were obtained as described under “Experimental procedures.” *D*, *left panel*, protein expression levels of Nav1.5, LITAF-HA, and tubulin. *Right panel*, respective changes of Nav1.5 protein levels normalized to tubulin. Although the theoretical molecular mass of the HA-tagged human LITAF is 20.6 kDa, our Western blotting data show a specific band at ∼28 kDa, which is likely due to post-translational modification (all values are means ± S.E., Student's *t* test, *n* = 11 each). *, *p* < 0.05.

To investigate any possible effect of LITAF on the Nav1.5 channel and its generated voltage-gated sodium current *I*_Na_, we used cultured 3-week-old rabbit cardiomyocytes (3wRbCM). We developed and used this model to study various ion channels underlying action potential duration (27, 28). For example, 3-week-old rabbit cardiomyocytes cultured for 48 h display a stable *I*_Na_ current (Fig. 1*A*). The cells were transduced with adenovirus encoding GFP and hemagglutinin (HA)–tagged LITAF. Overexpression of LITAF caused a significant increase (27.4%) in peak *I*_Na_ density (from −19.3 ± 2.2 pA/pF to −24.6 ± 2.21 pA/pF; *p* = 0.0073; Fig. 1*B*), yet there were no changes in voltage-dependent activation and inactivation kinetics (Fig. 1*C*). Western blotting results show that total Nav1.5 protein levels were significantly up-regulated (76.7%) in LITAF-overexpressing 3wRbCM (*p* < 0.05; Fig. 1*D*).

### LITAF controls Nav1.5 channel expression on the cell surface in HEK cells

**Figure 2:**
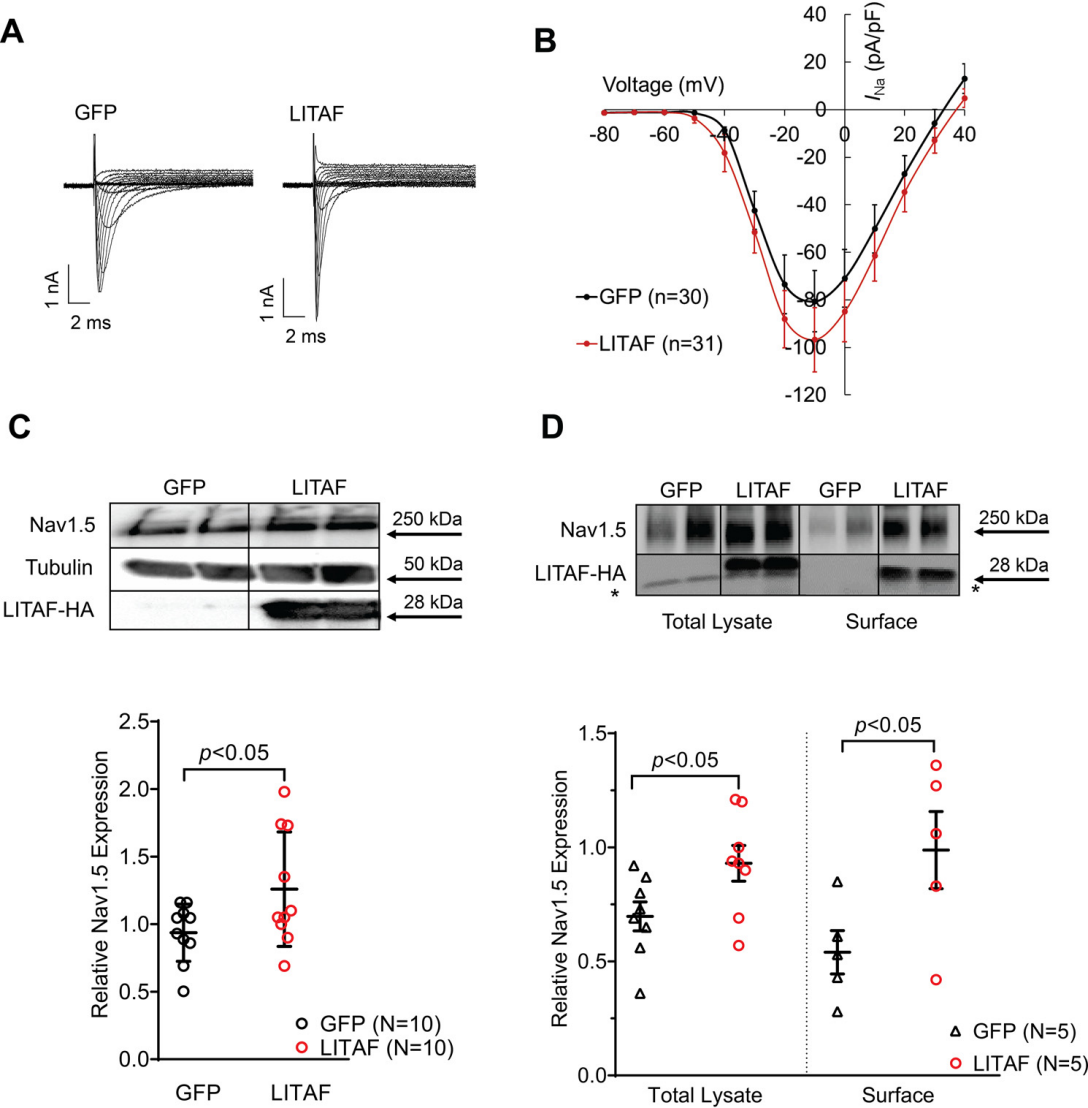
**LITAF increases *I*_Na_ as well as surface expression of Nav1.5 in stable HEK cells.** The cells stably co-expressing Nav1.5 and GFP were co-transfected with LITAF- and DsRed-expressing plasmids or control (GFP and DsRed). *A*, typical Na^+^ traces in GFP- and LITAF-expressing HEK cells. *B*, current–voltage relationship for *I*_Na_. *I*_Na_ was activated from −100 mV holding potential by depolarizing steps up to +40 mV in 10-mV increments. *C*, *top panel*, total expression of Nav1.5 and LITAF-HA. *Bottom panel*, respective change in Nav1.5 expression, normalized to tubulin (all values are means ± S.E.; Student's *t* test; *n* = 10). The uncropped probed membrane is shown in Fig. S1. *D*, total and surface protein expression of Nav1.5 and LITAF. Stable HEK cells were transfected with LITAF or GFP (control) expression plasmids. Cell-surface protein was biotinylated using sulfo-NHS-S-biotin, purified with NeutrAvidin beads from total cell lysates, subjected to SDS-PAGE, and blotted onto a polyvinylidene difluoride membrane. A representative immunoblot shows cell-surface and total lysate expression of Nav1.5, LITAF-HA, and tubulin (*top panel*) (the *asterisk* indicates an unspecific band). Respective changes in Nav1.5 expression, normalized to tubulin. All values are means ± S.E. (*n* = 5) (*bottom*).

Next, we switched to HEK cells as they are frequently used to study Nav1.5 *in vitro* (5, 29, 30). We used HEK cells that stably co-express Nav1.5 and GFP, to confirm our data obtained with 3wRbCM. HEK cells were co-transfected with expression plasmids for LITAF and red fluorescent protein (DsRed) or GFP and DsRed (control). Patch clamp results show that LITAF increased (20.1%) Nav1.5 current density from −80.6 ± 12.9 pA/pF to −96.8 ± 13.5 pA/pF; *p* < 0.05; Fig. 2*B*). Importantly, a significant concomitant increase in total Nav1.5 channel expression was noted (Fig. 2*C*). Surface biotinylation experiments were carried out to confirm that membrane expression of Nav1.5 was also significantly elevated upon LITAF overexpression (Fig. 2*D*), which is consistent with higher *I*_Na_ peak density in the presence of exogenous LITAF (Fig. 2*C*).

### Close-proximity interaction between LITAF and Nav1.5 channels

**Figure 3:**
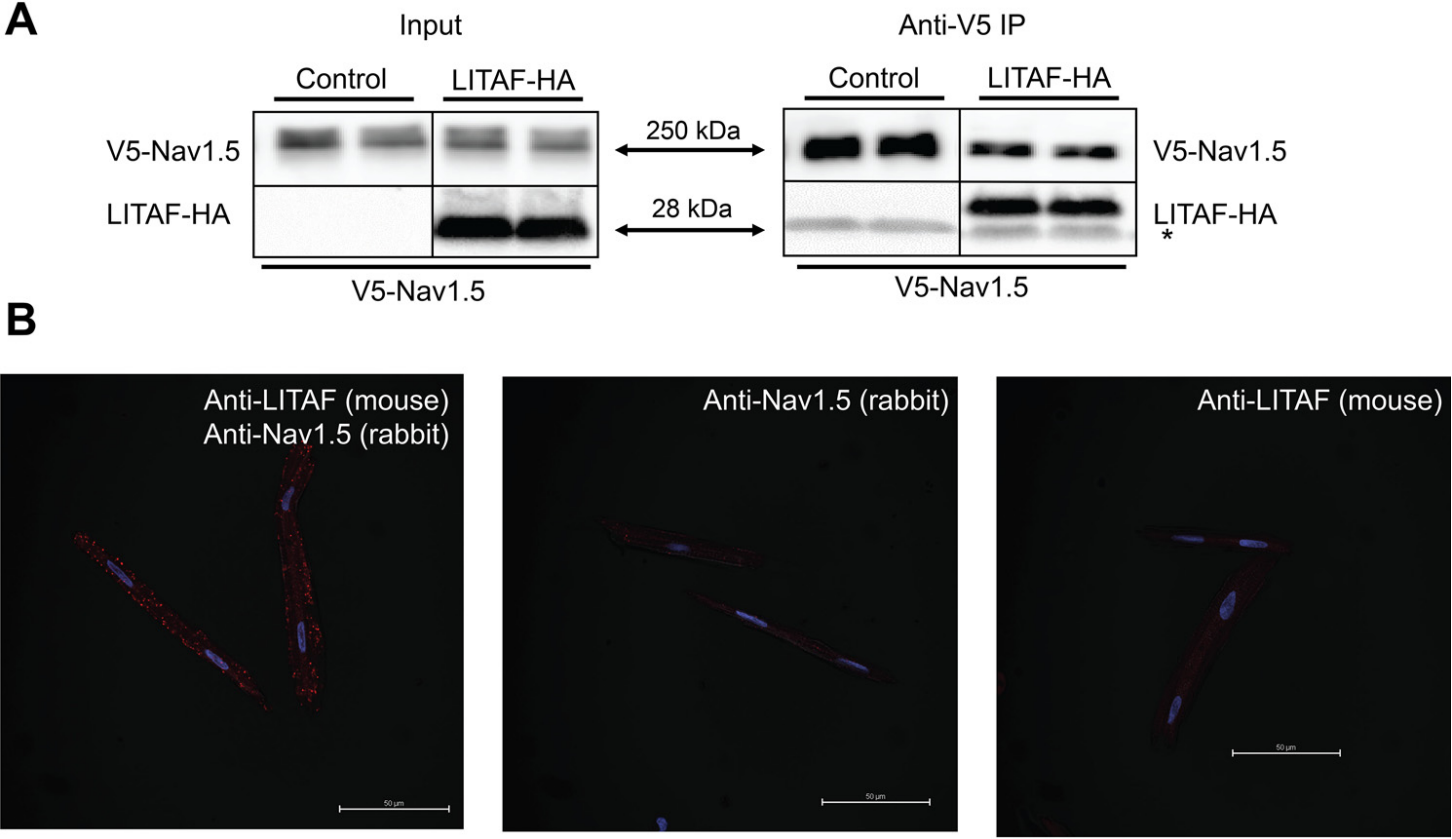
**Physical interaction between LITAF and Nav1.5 in HEK cells and 3wRbCM.***A*, V5 IP of lysates from HEK cells stably expressing V5-tagged Nav1.5 and transfected with plasmids for HA-tagged LITAF or empty expression plasmid (control) (*n* = 3). The *right panel* shows immunoprecipitated Nav1.5 and co-precipitated LITAF-HA and thus an interaction between LITAF and Nav1.5 (the *asterisk* indicates the light chain of the IP capture antibody). Input levels of Nav1.5 and LITAF-HA are shown in the *left panel*. *B*, Duolink *in situ* proximity ligation assay using mouse anti-LITAF and rabbit anti-Nav1.5 antibodies in 3wRbCM. The co-localization between molecules is indicated by *red puncta* (*left panel*). Virtually no puncta were detected in negative controls in which only one antibody was used, *i.e.* rabbit anti-Nav1.5 (*middle panel*) or mouse anti-LITAF antibodies (*right panel*). The nuclei were stained with DAPI (*blue*). Depicted merged confocal images (bright field, DAPI, and Texas Red) are representative of each condition. *Scale bar*, 50 μm.

Because our data suggested a functional interaction between LITAF and Nav1.5, we performed co-immunoprecipitation experiments on HEK cells stably expressing V5-tagged Nav1.5. The cells were co-transfected with plasmid encoding HA-tagged LITAF or empty control vector. Cell extracts were incubated with V5 antiserum, immunoprecipitated, separated by SDS-PAGE, transferred to membrane, and probed with anti-Nav1.5 antibody. Western blotting analyses suggest that LITAF is found in a protein complex with Nav1.5 (Fig. 3*A*). Additionally, we performed *in situ* PLA to look for any co-localization between these two molecules in 3wRbCM (Fig. 3*B*). The specificity of the assay was shown by the lack of staining using mouse anti-LITAF or rabbit anti-Nav1.5 as negative controls. The appearance of puncta with the combination of mouse anti-LITAF and rabbit anti-Nav1.5 supports proximity between LITAF and Nav1.5 in cardiomyocytes.

### LITAF blunts the NEDD4-2–dependent down-regulation of I_Na_ in HEK cells

**Figure 4:**
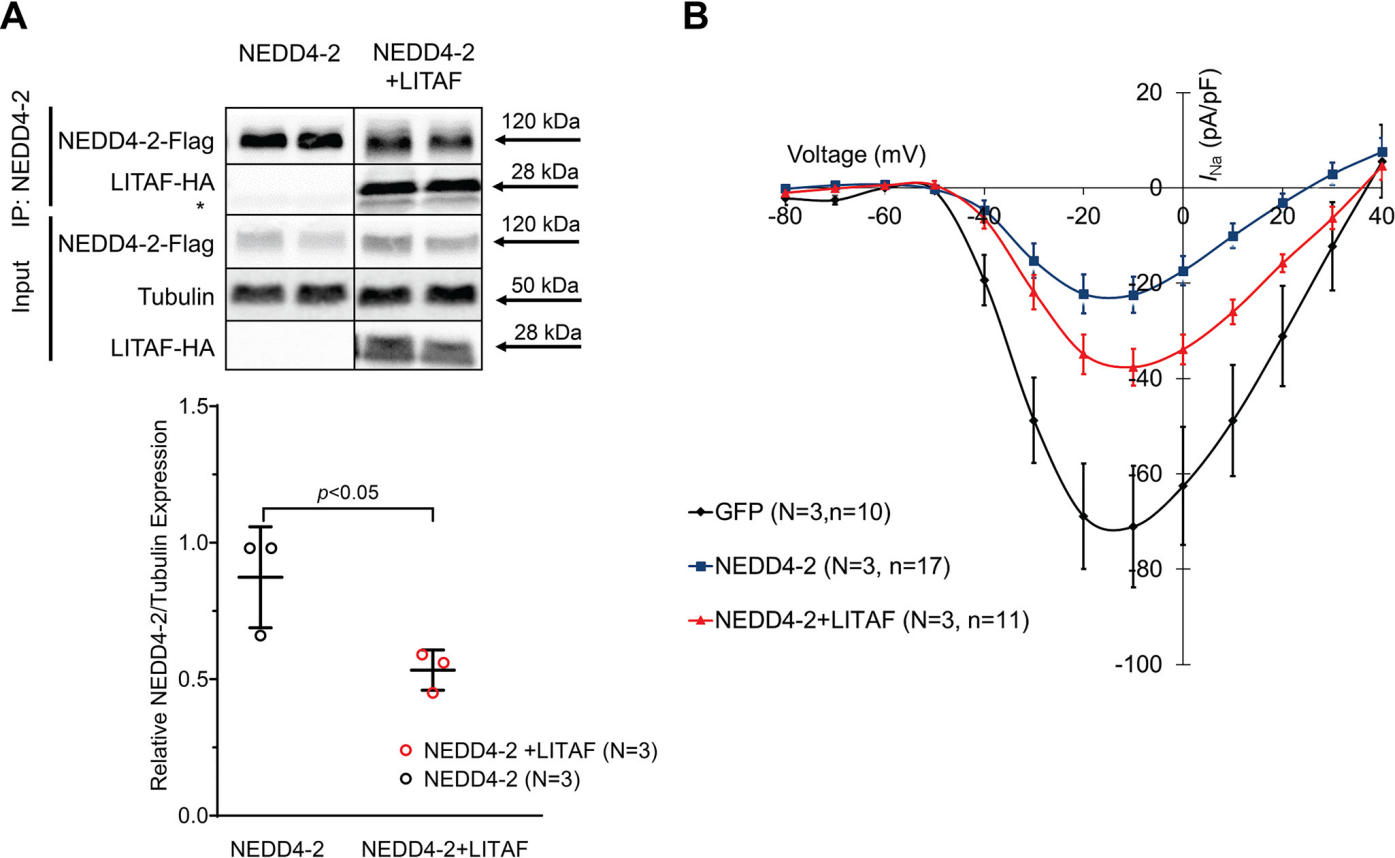
**LITAF partially abolishes NEDD4-2–dependent down-regulation of *I*_Na_ in HEK cells.***A*, co-immunoprecipitation of cell lysates from HEK cells transfected with plasmids for NEDD4-2–FLAG and LITAF-HA or just NEDD4-2–FLAG for 48h (*n* = 3). *Top panel*, the *top part* shows an interaction between immunoprecipitated NEDD4-2–FLAG and co-precipitated LITAF-HA (the *asterisk* indicates the light chain of the IP capture antibody), whereas the *bottom part* displays input levels of NEDD4-2–FLAG and LITAF-HA. *Bottom panel*, respective changes in input NEDD4-2 expression, normalized to tubulin. All values are means ± S.E. (*n* = 3). *B*, current–voltage relationships of *I*_Na_ peak currents for baseline conditions from cells expressing. GFP (control), NEDD4-2, or NEDD4-2 and LITAF (means ± S.E.). Two-way analysis of variance for repeated measures revealed significant differences in *I*_Na_ between all groups: GFP *versus* NEDD4-2: *F* = 80.05, *p* < 0.0001. NEDD4-2 *versus* NEDD4-2 + LITAF: *F* = 11.42, *p* = 0.0008. GFP *versus* NEDD4-2 + LITAF: *F* = 15.28, *p* = 0.0001.

NEDD4-2–dependent ubiquitination is a prerequisite for the degradation of surface Nav1.5 (4, 5). Because we have previously established physical and functional interactions between LITAF and the ubiquitin ligase NEDD4-1 (27), we entertained the possibility LITAF may also regulate NEDD4-2 with respect to Nav1.5. Therefore, we first looked for any physical interaction between NEDD4-2 and LITAF, which could be mediated by four WW domains of NEDD4-2 and two P*X*Y motifs of LITAF (17, 31). Total lysates of stable HEK cells transiently expressing HA-tagged LITAF and FLAG-tagged NEDD4-2 or FLAG-tagged NEDD4-2 were immunoprecipitated with FLAG antibody. The resulting immunoprecipitates were subjected to Western blotting. Fig. 4*A* shows co-precipitated LITAF indicating that LITAF and NEDD4-2 are found in the same protein complex. We also noticed that LITAF significantly reduced levels of co-expressed NEDD4-2 by 39% (Fig. 4*A*). Next, we wanted to assess the possible role of LITAF and NEDD4-2 in the homeostasis of Nav1.5. To this end, we transiently transfected HEK cells stably expressing Nav1.5 and GFP. Not surprisingly, co-expressed NEDD4-2 significantly decreased peak *I*_Na_ density (*e.g.* by 68%, viz. from −71.1 ± 12.7 pA/pF to −22.5 ± 3.8 pA/pF; −10 mV; *p* < 0.01; Fig. 4*B*), which is in agreement with a previous study by van Bemmelen *et al.* (5). Co-expressed LITAF, however, partially reversed the negative effect of NEDD4-2 on *I*_Na_ density (from −22.5 ± 3.8 pA/pF to −37.6 ± 13.0 pA/pF; −10mV; *p* < 0.01; Fig. 4*B*). Thus, this 52% recovery of *I*_Na_ in the presence of co-expressed LITAF is in line with the aforementioned 39% LITAF-dependent drop in NEDD4-2 levels. In summary, these functional data corroborate a role for LITAF modulating membrane expression of Nav1.5 by regulating NEDD4-2 ubiquitination-mediated degradation.

### Overexpression of LITAF results in ubiquitination and proteasomal degradation of NEDD4-2 in HEK cells

**Figure 5:**
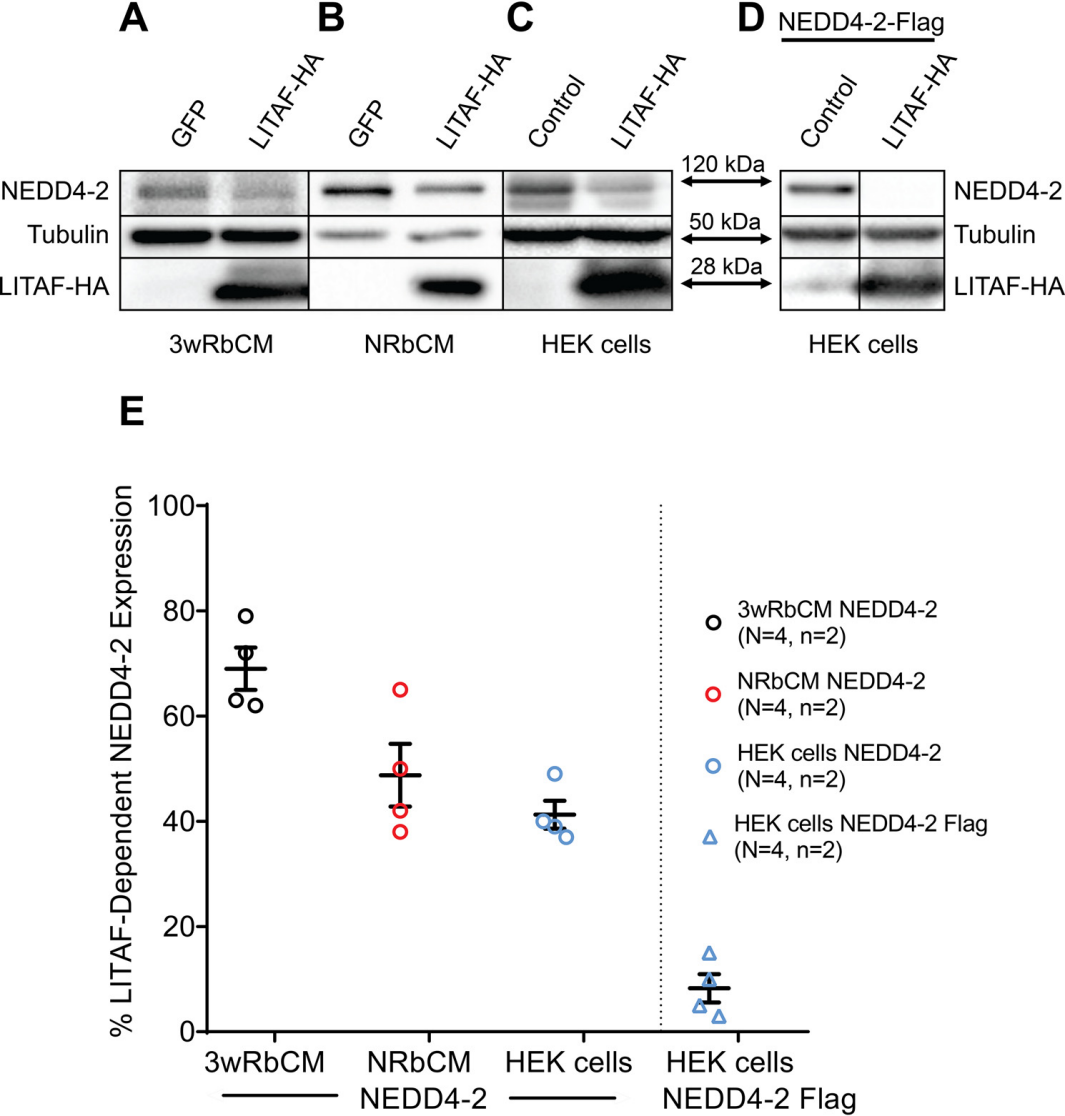
**LITAF down-regulates total NEDD4-2 expression.** Adenovirus for expression of GFP (control) or LITAF-HA was incubated with 3wRbCM (10 MOI) (*A*) or NRbCM (2 MOI) (*B*) for 48 h. HEK cells were transfected with plasmid encoding GFP (control) or HA-tagged LITAF (*C*) for 48 h. Representative Western blotting data from respective extracts to measure expression of NEDD4-2, tubulin, and LITAF-HA. *D*, NEDD4-2–FLAG, LITAF-HA, and tubulin expression in HEK cells transfected with expression plasmids for NEDD4-2–FLAG, LITAF-HA, or control plasmid for 48 h. *E*, Respective relative LITAF-dependent NEDD4-2 expression levels normalized to tubulin expression from four independent experiments performed in duplicate (Student's *t* test; *p* < 0.05). In the scatter plot, averaged values for the four independent experiments together with the means and S.E. values are shown.

Because the data presented in Fig. 4 suggest a functional interaction between LITAF and NEDD4-2 regulating Nav1.5 expression on the membrane, we wanted to investigate the possible role of LITAF in the regulation of NEDD4-2. To this end, we measured endogenous NEDD4-2 levels in 3-week-old and neonatal rabbit cardiomyocytes (NRbCM) overexpressing LITAF. We noted that LITAF overexpression reduced total levels of NEDD4-2 by ∼30% (3wRbCM) and 50% (NRbCM), respectively (Fig. 5, *A*, *B*, and *E*). Similarly, LITAF overexpression down-regulated endogenous NEDD4-2 levels by ∼60% in HEK cells (Fig. 5, *C* and *E*). Lastly, co-expression of LITAF and FLAG-tagged NEDD4-2 in HEK cells resulted in an ∼90% down-regulation of NEDD4-2–FLAG (Fig. 5, *D* and *E*).

**Figure 6:**
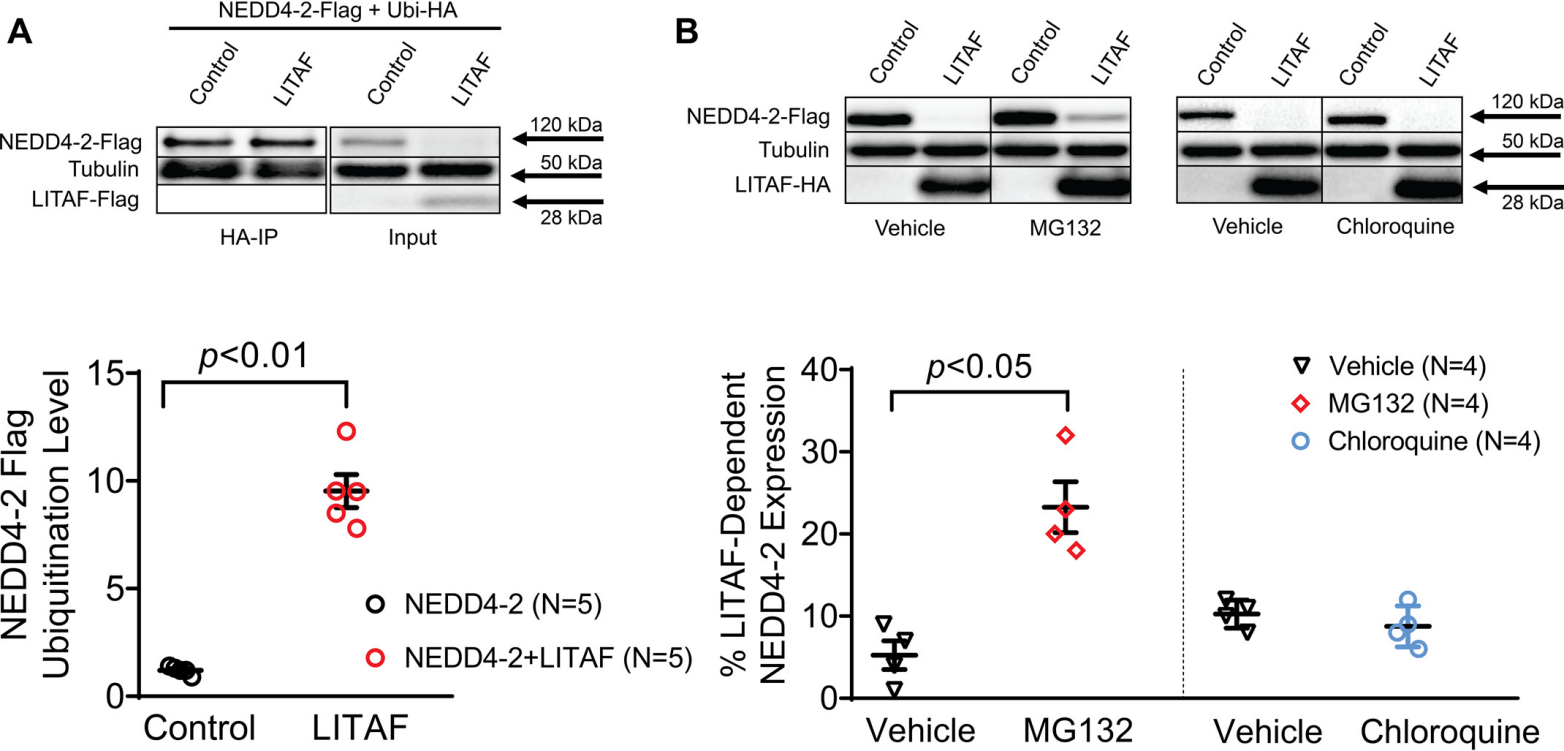
**LITAF causes ubiquitination and subsequent proteasomal degradation of NEDD4-2 in HEK cells.***A*, IP of lysates from HEK cells transfected with plasmids for FLAG-tagged NEDD4-2, HA-tagged ubiquitin, FLAG-tagged LITAF, or control plasmid for 48 h was performed with anti-HA antiserum. A representative immunoblot shows levels of ubiquitinated NEDD4-2–FLAG and tubulin and input levels of NEDD4-2–FLAG, LITAF-FLAG, or tubulin. Relative NEDD4-2–FLAG ubiquitination levels as calculated by the ratio of ubiquitinated NEDD4-2 normalized to ubiquitinated tubulin and total NEDD4-2 normalized to total ubiquitin levels (*n* = 5; means ± S.E.). In the scatter plot, averaged values for the five independent experiments together with the means and S.E. values are shown. *Bottom panel*, Student's *t* test, *p* < 0.01. *B*, LITAF-mediated degradation of NEDD4-2 through proteasomes. HEK cells were transfected with plasmids for NEDD4-2–FLAG, LITAF-HA or control plasmid for 24 h and then treated with vehicle (▿), 5 μm MG132 (), or 10 μm chloroquine () for 24 h. *Top panel*, representative Western blots show total expression of NEDD4-2–FLAG, LITAF-HA, and tubulin of treated cells. *Bottom panel*, respective relative expression levels (means ± S.E.) of total NEDD4-2 normalized to tubulin levels (*n* = 4, *n* = 2, *p* < 0.05).

We reasoned that LITAF overexpression likely caused ubiquitin-mediated degradation of NEDD4-2 in the various cell types tested. To test this hypothesis, we co-transfected expression plasmids for HA-tagged ubiquitin, FLAG-tagged NEDD4-2, FLAG-tagged LITAF, or control plasmid into HEK cells. Total cell extracts prepared 2 days later were immunoprecipitated with an anti-HA antibody to enrich for HA-ubiquitinated protein. The ubiquitinated protein fraction was separated by size using SDS-PAGE, transferred to a membrane, and probed against NEDD4-2. Western blotting data depicted in Fig. 6*A* indicate a significant, severalfold, LITAF-dependent increase in a single NEDD4-2 band implying likely monoubiquitination of the ubiquitin ligase. Next, we tested the pathways potentially responsible for LITAF-dependent NEDD4-2 degradation. We treated HEK cells expressing NEDD4-2, LITAF, or control plasmid with the selective proteasome inhibitor MG132 (32) or the lysosomal inhibitor chloroquine (33) for 24 h. Treatment of cells with MG132, but not treatment with chloroquine, partially prevented the LITAF-mediated down-regulation of NEDD4-2 (Fig. 6*B*). In summary, our data suggest that LITAF overexpression causes NEDD4-2 monoubiquitination and its subsequent degradation, in part through proteasomes. This, in turn, could account for the LITAF-mediated up-regulation of Nav1.5 expression and *I*_Na_ in cardiomyocytes.

### Computer modeling of LITAF overexpression

**Figure 7:**
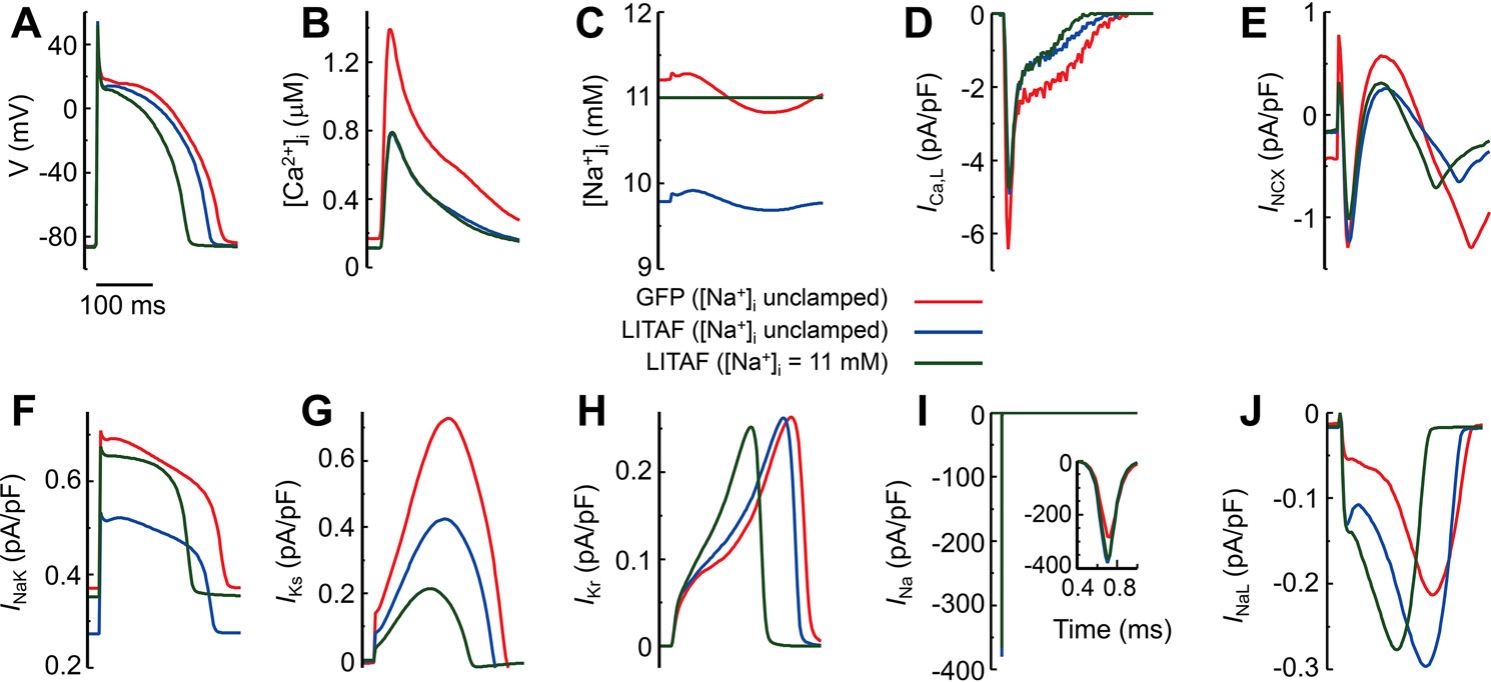
**Computer simulations of rabbit ventricular myocytes.** Cardiomyocytes were paced at 2.5 Hz under three different conditions: transduced with adenovirus encoding GFP with intracellular sodium concentration [Na^+^]*_i_* unclamped (*red traces*); adenovirally transduced causing LITAF overexpression with [Na^+^]*_i_* unclamped (*blue traces*); and adenoviral overexpression of LITAF with [Na^+^]*_i_* clamped to the same average steady-state 11 mm value reached under GFP (*green traces*). Transmembrane voltage *V* (*A*), cytosolic calcium concentration [Ca^2+^]*_i_*(*B*), [Na^+^]*_i_*(*C*), L-type calcium current *I*_Ca,L_ (*D*), Na^+^/Ca^2+^ exchanger current *I*_NCX_ (*E*), Na^+^/K^+^-pump current *I*_NaK_ (*F*), slowly activating delayed rectifier K^+^ current *I*_Ks_ (*G*), rapidly activating delayed rectifier K^+^ current *I*_Kr_ (*H*), fast sodium current *I*_Na_ (*I*), and late sodium current *I*_NaL_ (*J*).

Several genome-wide association studies (11, 12, 13) have implied a role for LITAF in QT interval and, therefore, in action potential regulation. Hence, we were interested in whether the LITAF-dependent effects on *I*_Na_ (in this study) and L-type calcium current *I*_Ca,L_ (27) could account for any changes in APD. To this end, we used a physiologically detailed model of rabbit ventricular myocyte with membrane voltage coupled to spatially distributed subcellular calcium dynamics. This model is im-proved from Moshal *et al.* (27) through several modifications. First, we included the late sodium current (*I*_NaL_) from Hwang *et al.* (34). Second, based on the voltage-clamp experiments, we modeled the effects of LITAF overexpression by increasing the conductance of both *I*_Na_ and *I*_NaL_ by 35% and reducing the number of LTCCs by 50% as in our previous study (27). We modeled the myocyte in current-clamp mode at 2.5 Hz (400-ms pacing cyclic length) and recorded the transmembrane voltage (*V*), the calcium transient, and several sarcolemmal currents after the intracellular sodium concentration [Na^+^]*_i_* reached steady state. We investigated three different conditions: control GFP with intracellular sodium concentration ([Na^+^]*_i_*) unclamped, LITAF overexpression with [Na^+^]*_i_* unclamped, and LITAF overexpression with [Na^+^]*_i_* clamped at a value of 11 mm corresponding to the average steady-state [Na^+^]*_i_* value under GFP; the latter condition was studied to dissect the effect of a change of steady-state [Na^+^]*_i_* on action potential duration. The comparison of GFP and LITAF simulations with unclamped [Na^+^]*_i_* (*red* and *blue traces* in Fig. 7) demonstrate that the 50% decrease of LTCC number (Fig. 7*D*) together with the 35% increase of sodium channel conductance (*I*_Na_ and *I*_NaL_ in Fig. 7, *I* and *J*) shortens the APD from 218 ms with GFP to 196 ms with LITAF (Fig. 7*A*). The results further show that this modest change of APD can be attributed to the subtle knock-on effect of *I*_Ca,L_ on other currents mediated by the changes in [Ca^2+^]*_i_* transient and [Na^+^]*_i_*, preliminarily explored in Moshal *et al.* (27). More specifically, the decrease in the [Ca^2+^]*_i_* transient amplitude (Fig. 7*B*) caused by the reduction of *I*_Ca,L_ (Fig. 7*D*) decreases the amplitude of both the forward and reverse modes of the Na^+^/Ca^2+^ exchanger current (*I*_NCX_), thereby causing a net decrease in [Na^+^]*_i_* caused by reduction of the net forward mode of *I*_NCX_ averaged over one pacing cyclic length. This decrease in [Na^+^]*_i_* in turn decreases the repolarizing Na,K-ATPase current (*I*_NaK_) (Fig. 7*F*), thereby partially counterbalancing the effect of *I*_Ca,L_ reduction on APD shortening. The results also show that although the magnitude of the rapid component of the delayed rectifier potassium current (*I*_Kr_) remains largely unchanged (Fig. 7*H*), the lowered action potential plateau significantly suppresses the fast component of the delayed rectifier potassium current (*I*_Ks_) (Fig. 7*G*), despite a modest APD shortening, thereby further contributing to counterbalancing APD shortening. Although both hyperpolarizing *I*_NaK_ and *I*_Ks_ are reduced under LITAF compared with GFP, the simulation results with [Na^+^]*_i_* clamped at the higher 11 mm steady-state value corresponding to GFP (*green traces* in Fig. 7) clearly show that the reduction of steady-state [Na^+^]*_i_* is the main causal mechanism underlying the smaller than expected APD shortening in the presence of significant *I*_Ca,L_ reduction. In particular, without this decrease, when [Na^+^]*_i_* is clamped at its higher GFP value, *I*_NaK_ is only slightly lower under LITAF than GFP (*red* and *green traces* in Fig. 7*F*), thereby yielding a more significant APD shortening (Fig. 7*A*). Importantly, APD is significantly shortened despite further reduction of *I*_Ks_ as a direct effect of membrane voltage (Fig. 7*G*), and peak *I*_NaL_ is only slightly reduced. An additional simulation carried out with unclamped [Na^+^]*_i_* and modeling only the effect of LITAF overexpression on *I*_Ca,L_, but not that on *I*_Na_ and *I*_NaL_, confirmed that *I*_Na_ and *I*_NaL_ increase has a minor effect on both APD and [Na^+^]*_i_* (results not shown). This is because, with LITAF overexpression, the reductions in *I*_NaK_ and *I*_Ks_ contribute more than the modest increase in *I*_NaL_ to counterbalancing the decrease of APD. Moreover, the fast component of *I*_Na_ makes a negligible contribution to [Na^+^]*_i_* because of its short ∼1-ms duration (*red* and *blue traces* in Fig. 7*I*), and the modest increase of *I*_NaL_ (Fig. 7*J*) is insufficient to counterbalance the decrease of [Na^+^]*_i_* caused by reduced *I*_NCX_ with LITAF overexpression. In summary, the computer modeling results demonstrate that changes in Ca^2+^ and Na^+^ homeostasis are primarily responsible for the more modest than expected APD shortening in the presence of LITAF overexpression. The decrease of functional *I*_Ca,L_ current density causes a decrease of Ca^2+^ transient amplitude that in turn decreases I_NCX_ and thus [Na^+^]*_i_* and *I*_NaK_. The decrease in *I*_Ca_,_L_ lowers the action potential plateau that in turn reduces the *I*_Ks_. Reduction of hyperpolarizing *I*_NaK_ and *I*_Ks_ then jointly contribute to counterbalancing the APD shortening because of decreased *I*_Ca,L_ with sodium current enhancement having a relatively small effect.

To investigate the role for LITAF on APD *in vivo*, we created a LITAF KO zebrafish line (c.45_55del), using Crispr/Cas9. Compared with WT fish, we observed a nonsignificant modest 14.4% increase in APD in homozygous KO zebrafish (mean ventricular APD80 ± S.E.: homozygous 301 ± 18.7 ms (*n* = 21) *versus* WT 263 ± 13 ms (*n* = 13); *p* = 0.16). Notably, there is a significant increase in the variations of the APD of the KO zebrafish as compared with WT fish (*p* < 0.05). Of note, we previously observed insignificant shortening of APD in 3-week-old rabbit cardiomyocytes overexpressing LITAF in agreement with computer modeling results (data not shown), whereas morpholino-mediated down-regulation of LITAF in zebrafish embryos resulted in prolongation of APD that did not reach statistical significance (27). This suggests that decreased [Na^+^]*_i_* and *I*_NaK_ as a knock-on effect of decreased *I*_Ca,L_ current density may be a common mechanism for the modest change of APD in both rabbit and zebrafish cardiomyocytes. Even though *I*_Ks_ assists *I*_NaK_ in counterbalancing the effect of decreased *I*_Ca,L_ on APD in rabbit cardiomyocytes, decreased [Na^+^]*_i_* and *I*_NaK_ still play a dominant role in keeping APD almost constant. Hence, even though *I*_Ks_ is not significantly expressed in zebrafish (35), decreased [Na^+^]*_i_* and *I*_NaK_ may suffice to counterbalance the effect of LITAF on *I*_Ca,L_ current density in zebrafish.

## Discussion

Previously, several genome-wide association studies have identified SNPs located upstream of the *LITAF* gene, encoding a regulator of endosomal trafficking (16, 17, 36), associated with QT interval (11, 12, 13). In the present study, we provide evidence that LITAF controls the voltage-gated sodium current *I*_Na_ in rabbit cardiomyocytes. The main finding of this study is that LITAF increases *I*_Na_ and expression of Nav1.5 channel on the membrane by promoting ubiquitination and degradation of the ubiquitin ligase NEDD4-2, which is indispensable for Nav1.5 turnover (4, 5). The voltage-gated sodium channel Nav1.5 is critical for the generation and conduction of cardiac action potentials. Mutations and changes in the expression level of Nav1.5 are associated with cardiac arrhythmias and sudden cardiac death as reviewed by Song *et al.* (37).

Because forward and retrograde trafficking of ion channels affect their overall function under physiological as well as pathological conditions, a large number of studies identified molecular factors involved in Nav1.5 trafficking and degradation (reviewed by Rook *et al.* (1)). For example, van Bemmelen *et al.* (5) showed that the HECT ubiquitin ligase NEDD4-2 could bind through its WW domains to the P*X*Y motif found at the C terminus of Nav1.5. Overexpression of NEDD4-2, but not the closely related NEDD4-1, increased Nav1.5 ubiquitination and subsequent degradation in HEK cells. Follow-up studies identified the ubiquitin-activating enzymes UBE1 and UBA6 (38), as well as the ubiquitin-conjugating enzyme UBC9 (39), to be required for NEDD4-2–dependent ubiquitination of Nav1.5 in neonatal rat cardiomyocytes and HEK cells. Our data suggest that this NEDD4-2–dependent control of Nav1.5 turnover is regulated by LITAF, previously identified as a protein involved in endosomal trafficking and inflammation (16, 17, 18, 19, 20, 21).

We provide evidence that 1) LITAF is found in protein complexes with Nav1.5 and NEDD4-2 in HEK cells and in 3wRbCM; 2) LITAF overexpression resulted in higher *I*_Na_ and total Nav1.5; 3) LITAF co-expression blunted the negative effect of NEDD4-2 on *I*_Na_; and 4) LITAF lowered exogenous and endogenous NEDD4-2 levels by promoting its ubiquitination and degradation. Thus, it is possible that LITAF and the small NEDD4 family-interacting proteins (NDFIPs), NDFIP1 and NDFIP2 (40) may share common mechanisms in regulating members of the NEDD4 family.

Interestingly, we have recently published that LITAF is also a regulator of L-type calcium channels in zebrafish and rabbit cardiomyocytes (27). Here, LITAF acts as an adaptor protein and activator of the HECT ubiquitin ligase NEDD4-1. This led to subsequent degradation of L-type calcium channel, thereby controlling its membrane levels, *I*_Ca,L_, and thus cardiac excitation. Currently, we can only speculate as to why LITAF regulates NEDD4-1 and NEDD4-2 differently with respect to their target molecules LTCC and Nav1.5 in cardiomyocytes. Similar to NDFIPs, we noted that LITAF overexpression lowered the amount of both NEDD4-2 (Fig. 5) and NEDD4-1,5 which was accompanied by increased ubiquitination (Fig. 6*A*).^5^ Importantly, LITAF also enhanced NEDD4-1-dependent ubiquitination of LTCC (27). It is conceivable that LITAF may act similarly to NDFIPs (40), possibly redirecting NEDD4-2 from the surface, where it would ubiquitinate Nav1.5, to another cellular location for sequestration or to target other substrates. Nevertheless, the net effect is to set a ratiometric relationship between LTCC and Nav1.5 at the membrane, thus regulating repolarization and its dynamics.

Although we have recapitulated LITAF's positive effect on *I*_Na_ in stable HEK cells (Fig. 2, *A* and *B*) and provided a molecular explanation for this phenomenon, *viz.* down-regulation of NEDD4-2 required for Nav1.5 degradation (Fig. 5), other mechanisms may contribute to the LITAF-dependent increase in *I*_Na_ in cardiomyocytes. For example, Luo *et al.* (41) have shown that elevated intracellular calcium levels increase NEDD4-2 mRNA expression in neonatal rat cardiomyocytes. Higher NEDD4-2 protein levels, in turn, reduced Nav1.5 and *I*_Na_. Because LITAF overexpression in rabbit cardiomyocytes resulted in lower *I*_Ca,L_ and calcium transients (27) but increased *I*_Na_ (Fig. 1), it is possible that resulting lower intracellular calcium levels could reduce NEDD4-2 mRNA expression, diminishing the negative impact NEDD4-2 has on surface Nav1.5 protein levels and *I*_Na_. Several studies reported lower Nav1.5 protein levels and *I*_Na_ in ischemia and heart failure (42, 43), likely increasing the risk for arrhythmias. In ischemia, no changes in Nav1.5 transcript levels were detected, implying accelerated degradation of Nav1.5 protein. At least one ubiquitin-dependent mechanism may account for the decrease in Nav1.5 levels in cardiac disease. Increased calcium concentrations in the cytosol caused by malfunctioning RYR2 increased NEDD4-2 expression lowering Nav1.5 levels, as data from volume-overload heart failure rat hearts suggested (41).

In agreement with the experiments associated with LITAF overexpression in 3-week-old rabbit cardiomyocytes and with LITAF KO in zebrafish (27), the computational simulation shows that LITAF overexpression shortens APD (Fig. 7). This effect takes place via the decrease in *I*_Ca,L_ resulting from the activation of HECT ubiquitin ligase NEDD4-1 (27). The consequence of decreased *I*_Ca,L_ is to reduce the Ca^2+^ transient amplitude that in turn reduces [Na^+^]*_i_*. Together with the lowered APD plateau caused by decreased *I*_Ca,L_, the decrease in [Na^+^]*_i_* reduces *I*_NaK_. Another effect of the lowered AP plateau is the decrease of *I*_Ks_. Reduction of hyperpolarizing *I*_NaK_ and *I*_Ks_ then jointly contribute to counterbalancing the APD shortening. The simulation also shows that the increase in sodium currents (*I*_Na_ and *I*_NaL_) caused by LITAF overexpression has a minor effect on [Na^+^]*_i_* and APD. The overall effect of LITAF overexpression on *I*_Na_ and *I*_Ca,L_ would lead to shorter APD and QT interval.

In summary, we conclude that LITAF is a novel regulator of Nav1.5 and *I*_Na_. The data provided in this study and the evidence of its complex role in regulating multiple ion currents make LITAF an interesting target for innovative strategies in the prevention and treatment of ventricular arrhythmias, not least those associated with reduced Nav1.5 function and *I*_Na_.

## Experimental procedures

### DNA

An expression cassette consisting of the cardiac CMV-enhanced 0.26-kb rat myosin late chain promoter (44), human β-globin intron, a fusion between the human LITAF ORF and three hemagglutinin (HA) tags, an internal ribosome entry site, the humanized *Renilla reniformis* GFP (hrGFP) ORF, and the human growth hormone polyadenylation signal (hGH1polyA) was cloned into pENTR 1A Dual (Thermo Fisher). The expression cassette was then transferred to the vector pAd/PL-DEST (Thermo Fisher) using the Gateway cloning system (Thermo Fisher). 293A cells (Thermo Fisher) were transfected with PacI-digested pAd/PL-DEST-CMV-MLC-LITAF-HA-hrGFP, and adenoviral stocks were prepared according to the manufacturer. As control, we created the vector pAd/PL-DEST-CMV-MLC-hrGFP that allows the expression of hrGFP in adenovirus. An expression cassette consisting of the CMV promoter, human β-globin intron, 3×HA LITAF, an internal ribosome entry site, hrGFP, and hGH1polyA was cloned into pENTR 1A Dual to obtain the mammalian expression vector pENTR-CMV-LITAF-HA-hrGFP. Similarly, the FLAG-tagged LITAF expression plasmid was created: pENTR-CMV-LITAF-FLAG-hrGFP. As control vector, pENTR-CMV-hrGFP–expressing hrGFP was created. The plasmid pcDNA3-Nedd4-2-FLAG was obtained from Dr. Sharad Kumar (Centre for Cancer Biology, University of South Australia and SA Pathology). pRK5-HA-ubiquitin-WT expressing HA-tagged ubiquitin (45) was purchased from Addgene (Addgene ID 17608). pDsRed-C1 vector was originally obtained from Clontech.

### Transfections

HEK cells and HEK cells stably co-expressing Nav1.5 and GFP were cultured in DMEM (Thermo Fisher) supplemented with 10% FBS. Transient transfections of HEK cells were performed for 48 h using Lipofectamine 2000 (Thermo Fisher) and following the manufacturer's instructions. Typically, we transfected 200 ng of pENTR-CMV-LITAF-HA-hrGFP (or pENTR-CMV-hrGFP), 200 ng of pcDNA3-NEDD4-2–FLAG, 200 ng of pcDNA3, and 1.8 µl of Lipofectamine 2000 per 12-well (Figs. 5, *C* and *D*, and 6B) or 500 ng of pRK5-HA-Ubiquitin-WT, 500 ng of pENTR-CMV-LITAF-FLAG-hrGFP (or pENTR-CMV-hrGFP), 500 ng of pcDNA3-Nedd4-2-FLAG, 1.5 µg of pcDNA3 (Invitrogen), and 7.2 µl of Lipofectamine 2000 per 6-cm dish (Fig. 6*A*). For transient transfections of HEK cells stably expressing Nav1.5, we generally used 1600 ng of pENTR-CMV-LITAF-HA-hrGFP (or pENTR-CMV-hrGFP), 1600 ng of pcDNA3-NEDD4-2–FLAG (or pcDNA3), 800 ng of pENTR-CMV-hrGFP, 270 ng of pDsRed-C1, and 10 µl of Lipofectamine 2000 (Figs. 2, *B–D*; 3*A*; and 4, *A* and *B*).

### Preparation of rabbit cardiomyocytes

All animal experiments and procedures were approved by the Rhode Island Hospital Institutional Animal Care and Use Committee (reference nos. 0188-14 and 5013-17). 3-week-old ventricular cardiomyocytes were isolated from the hearts of 3-week-old NZW rabbits (both sexes) with standard enzymatic techniques using a Langendorff system. NZW rabbits were administered pentobarbital sodium (65 mg/kg IP) and heparin (1,000 units/kg IP). The filtered cells were maintained in 45 mm KCl, 65 mm potassium glutamate, 3 mm MgSO_4_, 15 mm KH_2_PO_4_, 16 mm taurine, 10 mm HEPES, 0.5 mm EGTA, and 10 mm glucose (pH 7.3) for half an hour. In five subsequent steps, the Ca^2+^ concentration was increased to 1.8 mm. The cells were centrifuged, resuspended in DMEM supplemented with 7% FBS and antibiotics, plated on laminin-coated cover glasses or tissue culture dishes. After 2–3 h, the medium was replaced and adenovirus (50 MOI) added to the cells. The cells were maintained at 37 °C with 5% CO_2_, and ∼48 h later, the cells were used for patch clamping and biochemistry. The isolation of neonatal rabbit cardiomyocytes was exactly performed as described (27).

### Electrophysiological recordings

3-week-old rabbit cardiomyocytes were transduced with adenovirus ∼48 h before *I*_Na_ recording. The experiments were conducted at 34–36°C with an Axopatch-200B amplifier, Digidata 1440A, and pClamp 10 software (Axon Instruments). The signal was acquired at 20 kHz and filtered at 10 kHz. The whole-cell configuration was obtained in standard Tyrode solution containing 140 mm NaCl, 5.4 mm KCl, 0.33 mm NaH_2_PO_4_, 1.8 mm CaCl_2_, 1 mm MgCl_2_, 10 mm HEPES, 5.5 mm d-glucose, and pH 7.4 was adjusted with NaOH. The pipette solution contained 80 mm CsCl, 80 mm Cs aspartate, 1 mm MgCl_2_, 1 mm CaCl_2_, 11 mm EGTA, 10 mm HEPES, 5 mm Na_2_ATP, and pH 7.3 was adjusted with CsOH. The pipette resistance was 1–2 MΩ. After obtaining the whole-cell configuration and compensation of the capacitive artifact and access resistance by 80%, we replaced the Tyrode solution with low sodium solution containing 100 mm *N*-methyl-d-glucamine, 20 mm tetraethylammonium chloride, 10 mm NaCl, 5 mm CsCl, 2 mm CaCl_2_, 1.2 mm MgCl_2_, 10 mm HEPES, 5 mm d-glucose, 0.01 mm nifedipine, and pH 7.4 was adjusted with HCl. To obtain *I*–*V* curves for *I*_Na_, the cells were depolarized from −100 mV holding potential to +60 mV by 200-ms depolarizing steps in 10-mV increments. The *I*–*V* curve in Fig. 1*B* was fitted with OriginPro 2019 Boltzmann *I*–*V* function, and the obtained *E*_rev_ values have been used to calculate conductance as *g* = *I*_Na_/(*V* − *E*_rev_) at different potentials for the activation curve in Fig. 1*C*. To study inactivation of *I*_Na_, the 200-ms depolarizing steps were followed by a 220-ms step to −20 mV. The *I*_Na_ peak values were always measured relatively to the tail current at the end of the corresponding voltage pulse. To study effects of LITAF and NEDD4-2 on *I*_Na_ in stable HEK cells, we used the same solutions and voltage protocols as described above for 3-week-old rabbit cardiomyocytes.

### Immunoblot analysis

Surface biotinylations, co-IP, and immunoblots were exactly carried out as in previous studies (14, 15, 27).

### In situ proximity ligation assay

For PLA, 3wRbCM were plated on 12-mm laminin-coated circular glass coverslips and cultured for 3–5 h in 24-well plates. The cells were fixed with 4% paraformaldehyde at room temperature for 15 min followed by permeabilization at room temperature with 0.1% Triton X-100 for 10 min prior to the assay. PLA was performed according to the manufacturer's instructions (Sigma: Duolink® *in situ* Red starter kit mouse/rabbit). After five washes with PBS, the cells were blocked for 30 min at 37 °C. Antibodies used for PLA were as follows: rabbit anti-Nav1.5 (Alomone; ASC-005; 1:100) and mouse anti-LITAF (Abnova; H00009516-B01P; 1:100). The images were captured using a Nikon A1R laser scanning confocal microscope, DAPI (excitation, 359; emission, 461), Texas Red (excitation, 595; emission, 612) filters for detection purposes at 60× zoom, and Elements software (Nikon).

### Rabbit ventricular myocyte model

To study the effect of LITAF in myocytes, we used a physiologically detailed rabbit ventricular myocyte model of Moshal *et al.* (27). This model is based on earlier models developed by Restrepo *et al*. (46) and further improved by both Terentyev *et al.* (47) and Zhong *et al.* (48). Combining together a large number of ∼16,000 diffusively coupled Ca^2+^ release units, this multiscale model successfully links the whole-cell level Ca^2+^ dynamics to the local subcellular Ca^2+^ dynamics in each Ca^2+^ release unit, which incorporates four LTCC and 100 ryanodine receptors, both implemented by Markov models. By including the Ca^2+^-dependent channels LTCC and Na^+^-Ca^2+^ exchanger and other sarcolemmal currents, this detailed model describes the bidirectional coupling of Ca^2+^-*V*_m_ dynamics, which is essential in cardiac electrophysiological behavior. The details of LTCC model and ryanodine receptor model are shown by Zhong *et al.* (48).

We carried out the current clamp simulations by pacing the myocytes at 2.5 Hz (400 ms) for three different conditions: control GFP with Na^+^]*_i_* unclamped, LITAF overexpression with [Na^+^]*_i_* unclamped, and LITAF overexpression with [Na^+^]*_i_* clamped at a value of 11 mm corresponding to the average steady-state [Na^+^]*_i_* value under GFP. For the [Na^+^]*_i_* unclamped simulations, we collected the data after the7 intracellular sodium concentration reached the steady state.

Based on Moshal *et al.* (27), we applied the modifications described by the following paragraphs. All the modified parameters are listed in Table 1.

**Table 1:** Modified parameters in the rabbit ventricular myocyte model Several parameters were changed in this refined model compared to the one described by Moshal *et al.* (27).

Parameter	Definition	Value
		*mS*/μ*F*
$g_{NaL}$	*I*_NaL_ conductance (GFP)	0.01
$g_{NaL}$	*I*_NaL_ conductance (LITAF)	0.0135
$g_{Na}$	*I*_Na_ conductance (GFP)	12.0
$g_{Na}$	*I*_Na_ conductance (LITAF)	16.2

Following Hwang *et al.* (34), we included the late sodium current (*I*_NaL_). The model is described by the following set of equations.

(Eq. 1) $$E_{Na}=\frac{RT}{F}\log\left(\frac{{\left[Na\right]}_{o}}{{\left[Na\right]}_{i}}\right)$$

(Eq. 2) $$m_{l,\infty }=\frac{1}{1+\exp\left(-\frac{V+52.728}{24.327}\right)}$$

(Eq. 3) $$h_{l,\infty }=\frac{1}{1+\exp\left(-\frac{V-9.2715}{19.913}\right)}$$

(Eq. 4) $$I_{NaL}=g_{NaL}m_{l,\infty }^{3}h_{l,\infty }^{2}(V-E_{Na})$$

Based on the voltage-clamp experiments, we modeled the effects of LITAF overexpression by increasing the conductance of both *I*_Na_ and *I*_NaL_ by 35% in addition to reducing the number of LTCCs by 50% as in the work of Moshal *et al.* (27).

### Aquaculture

The experiments were performed on zebrafish (*Danio rerio*) on the AB/Tuebingen (AB/Tu) background in accordance with animal protocols approved by the Harvard Medical School Institutional Animal Care and Use Committee. Care and breeding of zebrafish were performed as described previously at 28.5 °C, and embryos were maintained in standard E3 medium (49).

### Generation of LITAF knockout in zebrafish

WT AB/Tu zebrafish were crossed, and the resultant embryos were injected with a solution containing crRNA targeting LITAF (ATGGAGAACACGACCCTTGTGGG), Alt-R® tracrRNA, and Alt-R® S.p. HiFi Cas9 Nuclease V3 (all from Integrated DNA Technologies), according to the manufacturer's instructions. These F0 embryos were raised up and outcrossed individually to WT AB/Tu fish. Resultant F1 embryos were sequenced to identify clutches with frameshift mutations that are predicted to be loss-of-function mutations. Selected F1 clutches were raised, fin-clipped, and sequenced to confirm the mutation in each individual fish. Adult fish heterozygous for an 11-bp deletion in exon 2 of LITAF (c.45_55del), which is predicted to result in a severely truncated protein of 22 amino acids (p.L16PfsX8), were in-crossed. The resultant F2 embryos were WT, heterozygous, and homozygous for the 11-bp deletion in expected Mendelian ratios and were used for downstream experiments.

### Optical mapping of isolated zebrafish hearts

Optical mapping and signal processing were performed as previously described (49). Briefly, the hearts were isolated from zebrafish embryos at 72 h postfertilization and stained with the transmembrane potential–sensitive dye in the FluoVolt^TM^ membrane potential kit (Thermo Fisher). The resultant fluorescence intensities were recorded with a high-speed CCD camera (RedShirtImaging), and images were analyzed using custom scripts in MATLAB.

### Statistical analysis and curve fitting

Statistical analysis and curve fitting were performed with GraphPad Prism 8 and OriginPro 2019. The data are presented as means ± S.E. A difference was considered significant at *p* < 0.05.

### Replicates

Throughout the study, we used biological replicates, *i.e.* different animals or different frozen HEK cell stocks (as indicated by *N*), and technical replicates (as indicated by *n*).

## Data availability

All of the data are contained within the article.

## Footnotes

5 N. N. Turan, K. S. Moshal, K. Roder, B. C. Baggett, A. Y. Kabakov, S. Dhakal, R. Teramoto, D. Y.-E. Chiang, M. Zhong, A. Xie, Y. Lu, S. C. Dudley, Jr., C. A. MacRae, A. Karma, and G. Koren, unpublished data.
