# Supplementary material for: The endosomal trafficking regulator LITAF controls the cardiac Nav1.5 channel via the ubiquitin ligase NEDD4-2
Source: J Biol Chem. 2021 Jan 13;295(52):18148–59. doi: 10.1074/jbc.RA120.015216 (PMC7939464; doi:10.1074/jbc.RA120.015216)
Supplement: Supplementary file 1 [file mmc1.pdf]

SUPPLEMENT DATA

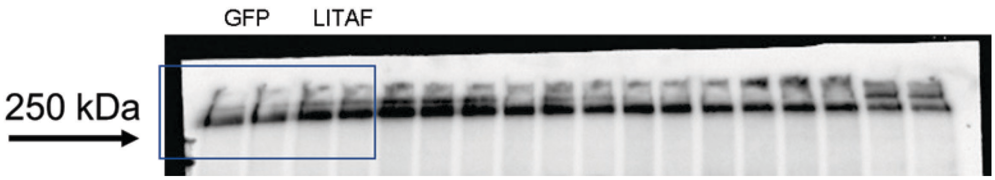

FIGURE S1. Source data for Figure 2C. Full-sized image of panel 2C, which is depicted as boxed.
